# The role of TMEM26 in disrupting tight junctions and activating NF-κB signaling to promote epithelial-mesenchymal transition in esophageal squamous cell carcinoma

**DOI:** 10.1016/j.clinsp.2023.100276

**Published:** 2023-08-21

**Authors:** Guohu Han, Shuangshuang Zhou, Junjun Shen, Yuanyuan Yang, Xuyu Bian, Yahu Li, Rui Ling, Rongrui Liang, Min Tao

**Affiliations:** a Department of Oncology, Dushu Lake Hospital Affiliated to Soochow University, Suzhou, Jiangsu, China; b Department of Oncology, Jingjiang People's Hospital Affiliated with Yangzhou University, Jingjiang, Jiangsu, China; c Department of Oncology, Affiliated Hospital of Jiangsu University, Zhenjiang, Jiangsu, China; d Department of Oncology, The First Affiliated Hospital of Soochow University, Suzhou, Jiangsu, China

**Keywords:** Tight junctions, Cell movement, Epithelial cells, Esophageal squamous cell carcinoma

## Abstract

**Objectives:**

Metastasis is one of the biggest challenges in the management of Esophageal Squamous Cell Carcinoma (ESCC), of which molecular mechanisms remain elusive. The present study aimed to explore the roles and underlying mechanisms of Transmembrane protein 26 (TMEM26) in ESCC.

**Method:**

TMEM26 expressions in tumorous and adjacent tissues from patients with ESCC and in normal esophageal epithelial and ESCC cell lines were detected by immunostaining and western blotting, respectively. The Epithelial-Mesenchymal Transition (EMT), a critical process during metastasis, was investigated by wound healing and Transwell assays, and EMT-related proteins were examined after the TMEM26 alteration in ESCC cell lines. NF-κB signaling activation and Tight Junction (TJ) protein expression were analyzed by western blotting and immunofluorescence, respectively. *In vivo* verification was performed on the liver metastatic murine model.

**Results:**

Compared with non-cancerous esophageal tissues and cells, the TMEM26 expression level was higher in ESCC samples and cell lines, where the plasma membrane localization of TMEM26 was observed. The EMT-related processes of ESCC cells were suppressed by RNAi depletion of TMEM26 but aggravated by TMEM26 overexpression. Mechanistically, TMEM26 promoted NF-κB signaling to accelerate EMT in ESCC cells. The plasma membrane presentation and assembly of TJ proteins were impaired by TMEM26.

**Conclusion:**

Overall, TMEM26 acts as a critical determinant for EMT in ESCC cells by disrupting TJ formation and promoting NF-κB signaling, which may be a potential therapeutic target for treating metastatic ESCC.

## Highlights

• The involvement of TMEM26, a novel plasma membrane protein, in cancer EMT was examined for the first time.
• The discovery of TMEM26 and its regulation on the tight junction activates NF-κB signaling as a novel therapeutic target of metastatic ESCC.
• A mechanism that directly controls the assembly of tight junctions at the plasma membrane, which affects a plethora of signaling pathways, was discovered.

## Introduction

Esophageal Carcinoma (EC) is one of the most frequently diagnosed tumors in the digestive tract and is mainly classified into two types of EC: Esophageal Squamous Cell Carcinoma (ESCC) and Esophageal Adenocarcinoma (EAC). ESCC is the predominant subtype of EC in Asians, which is mainly caused by smoking, alcohol consumption, hot drinks, and other poor dietary habits.^1^ ESCC is characterized by low differentiation, which drives the malignant transformation of esophageal epithelial cells to initiate its pathogenesis.^2^

Another known characteristic of ESCC is the high rate of metastasis.^3^ In general, metastatic ESCC at an early stage can be treated with surgery combined with chemotherapy and radiation therapy. Unfortunately, most ESCC cases are diagnosed at an advanced stage; hence, the prognosis is always poor.^2^^,^^4^ Despite the surgical technique and perioperative management advances, recurrence, and distant metastasis of ESCC are frequently reported.^5^ Therefore, the mechanisms orchestrating ESCC metastasis should be understood, where the Epithelial-Mesenchymal Transition (EMT) program plays a critical role.

EMT is an important process for cancer cells to acquire a metastatic phenotype 6, 7, 8. A plethora of signaling pathways is involved in the control of EMT in ESCC. For example, cell division cycle-associated 7, the gene amplified in ESCC, enhanced the metastasis and invasion of ESCC cell lines both *in vivo* and *in vitro* by activating TGF-β signaling to facilitate EMT.^9^ Kinesin Family member C3 (KIFC3), a kinesin superfamily protein, was upregulated in ESCC tissues and associated with poor prognosis in patients with ESCC, and mechanistic studies indicated that KIFC3 promoted proliferation, migration, and invasion of ESCC via β-catenin signaling and EMT.^10^ YAP activation driven by C12orf59, a novel cancer-related factor prominently higher in both tumor tissues and most ESCC cell lines, contributes to the EMT of ESCC, and thereby, combined treatment of C12orf59, and YAP inhibitors could be developed as a therapeutic strategy for metastatic ESCC.^11^ Furthermore, a recent study revealed that neuron-specific gene family member 1 may amplify the ERK signaling pathway to promote ESCC cell EMT.^12^ Hence, uncovering the molecules driving the EMT process can help identify therapeutic strategies and prolong the survival of patients with metastatic ESCC.

Epithelial cells contain several classes of cell-cell junctions: adherens junction, Tight Junction (TJ), desmosomes, and gap junction. TJ consisted of multiple proteins, such as claudin, occludin, and Zonula Occludens-1/2/3 (ZO-1/2/3). TJ organizes the junctional complex on the apical side between neighboring epithelial cells and plays supportive roles in cell architecture. TJs are also important for maintaining cell polarity and substance passing between epithelial cells.^13^ Increasing evidence supports the well-established functional role of TJ proteins in cancer pathogenesis.^14^ The intracellular regions of TJ proteins bind to cytoskeletal elements and signaling molecules, acting downstream of certain essential signaling pathways to regulate cell migration, proliferation, and differentiation, all of which are important cancer hallmarks essential for tumor growth and metastasis.^15^

To initiate the EMT process, the contact between epithelial cells must be broken down, including the disruption of TJ structures. TJ disassembly promotes the loss of cell polarity and reorganization of the cytoskeleton to potentiate cell migration and cancer metastasis.^13^ Several studies reported the role of TJ in the EMT of cancer cells.^13^^,^^14^ The lncRNA-NEAT1 was induced by the carcinogen arecoline to downregulate ZO-1 expression and destabilized TJ assembly, which enabled the EMT initiation in head and neck cancer cells.^16^ The flavonoid compounds, baicalin, and baicalein, disrupted the TJ structure by targeting the first PDZ domain of ZO-1 to suppress its interaction with occludin, resulting in EMT-like morphological changes in the Madin–Darby canine kidney II cells.^17^ In colorectal cancer cells, the tumor-related mutant β-catenin downregulated the expression of ZEB1 transcription factor-controlled TJ proteins, claudin-7, and E-cadherin, to impair the TJ structure, resulting in increased cell motility and EMT progression.^18^ However, whether TJ impairment is involved in the EMT of ESCC remains to be elucidated. Moreover, whether a membrane protein could directly impair the TJ structure (without affecting the TJ protein expression) to promote EMT progression remains elusive.

The Transmembrane protein 26 (TMEM26) gene encodes a protein containing multiple transmembrane fragments. It was detected specifically in brown and beige adipocytes and was, therefore, considered the marker for these cells.^19^ Information on the function of this putative plasma membrane protein in the literature is extremely limited. Intrigued by the novelty of this gene and its possible involvement in ESCC, this study aimed to evaluate the role of TMEM26 in ESCC tissues and a series of ESCC cell lines. Immunostaining indicated elevated TMEM26 expression in ESCC tumors. Various ESCC cell lines showed a high TMEM26 expression, where its plasma membrane localization was confirmed. The RNAi depletion of TMEM26 in TMEM26-high ESCC cells suppressed EMT-related alterations, including invasion, migration, and marker gene expression. Conversely, TMEM26 overexpression in TMEM26-low ESCC cells promoted these EMT-related alterations. Interestingly, TMEM26 depletion or overexpression did not affect cell growth, indicating its specific involvement in the EMT process. The animal study demonstrated the contributive role of TMEM26 in metastatic ESCC. Mechanistically, TMEM26 promoted NF-κB signaling to accelerate EMT in ESCC cells. The plasma membrane presentation and TJ protein assembly were impaired by TMEM26, which was likely to be another mechanism for EMT regulation by TMEM26 in ESCC. Therefore, TMEM26 disrupted TJ formation and promoted NF-κB signaling during the EMT activation in ESCC. The development of TMEM26-targeting drugs might offer an innovative line of therapies for ESCC.

## Materials and methods

### Cell lines, reagents, and antibodies

ESCC cell lines (KYSE150, KYSE270, KYSE450, TE4, and TE8) and a normal esophageal epithelial cell line (HET-1A) were obtained from ATCC (MA, USA). The Roswell Park Memorial Institute (RPMI)-1640 medium, 5-diphenyltetrazolium bromide (MTT) kit, and 4′,6-Diamidino-2-Phenylindole (DAPI) stain solution were supplied by Sigma (MO, USA). Lipofectamine 3000 reagents and Bicinchoninic Acid (BCA) kit were purchased from Thermo Scientific (CA, USA). Fetal Bovine Serum (FBS) was procured from GIBCO (CA, USA). Horseradish Peroxidase (HRP)-conjugated goat anti-rabbit/mouse antibodies were obtained from Cell Signaling Technology (MA, USA). Alexa Fluor 488 or 555 donkey anti-rabbit/mouse secondary antibodies were purchased from Beyotime (Shanghai, China). Information on primary antibodies used in this study is listed in Table S1.

### Patients and ESCC specimen collection

ESCC biopsy specimens and non-cancerous biopsy samples from the same patients (*n* = 40) were collected from Jingjiang People's Hospital Affiliated with Yangzhou University. All donors provided signed written informed consent. The experimental protocol was approved by the ethics committee of Jingjiang People's Hospital Affiliated with Yangzhou University.

### Immunohistochemistry

ESCC tumor specimens were incubated at 60 °C for 2 h at pretreatment with dimethyl benzene for dewaxing. The sections were then hydrated with a gradient of alcohol and washed with double distilled water (ddH_2_O). Subsequently, the sections were fixed with 3% hydrogen peroxide for 15 min and then washed with Phosphate-Buffered Saline (PBS) three times at Room Temperature (RT). Then, the sections were incubated with primary TMEM26 antibodies at 37 °C for 30 min and at 4 °C overnight. Afterward, the secondary antibody was added and incubated for 30 min at 37 °C. Next, the sections were sequentially incubated with HRP-labeled avidin for 30 min at 37 °C and reacted with domain antibodies for 3–10 min before the reaction was stopped by ddH_2_O. Counterstaining was performed with hematoxylin and then dehydration. The sections were finally examined under a microscope (BX51, Olympus, Japan).

### The liver metastatic murine model

The Institutional Animal Care and Use Committee at the Affiliated Hospital of Jiangsu University approved all animal procedures in this study. Twenty BALB/c nude male mice were purchased from Cavens Laboratory Animal Inc. (http://www.cavens.com.cn/). All mice were maintained under specific-pathogen-free conditions and subjected to 1-week acclimation before starting animal experiments.

To evaluate the ESCC cell metastasis to the liver, the mice were divided into four groups: si-NC, si-TMEM26 #2, vector, and TMEM26 OE. Briefly, ESCC cells (KYSE270 and KYSE150) with either TMEM26 knockdown or overexpression were injected into the portal vein of the mouse liver. ESCC cells with si-NC transfection or vector injected into the portal vein of the liver served as the control. At 28 days post-injection, liver tissues were dissected, and subjected to hematoxylin and eosin staining to examine liver metastasis.

### Cell culture and transfection

KYSE150, KYSE270, KYSE450, TE4, and TE8 cells were cultured in a 10 cm dish with RPMI-1640 and 10% FBS in an incubator at 37° and 5% CO_2_. siRNA transfection (with sequences listed in Table S2) was conducted using Lipofectamine 3000.

### MTT assay for cell viability

The cells (1 × 10^4^ cells/well) were grown overnight in 96-well plates. The volume of the culture medium was 100 μL per well. After overnight incubation, 10 μL of MTT solution was pipetted into each well and incubated for another 4 h. Subsequently, the medium was discarded, and formazan crystals were dissolved by adding 150 μL of dimethyl sulfoxide. The absorbance was read at 570 nm wavelength using a microplate instrument (Thermo Scientific).

### Wound healing assay

The assay was performed with the CytoSelec 24-well Wound Healing Assay Kit (BIOCAT GmbH, Heidelberg, Germany) following the instruction provided with the kit. Briefly, 250 µL of cell suspension (2 × 10^5^ cells/mL) was added on each side of the insert. Cells were cultured until they formed a monolayer around the insert (about 24 h). Next, the inserts were removed from the wells, leaving a precise open “wound field” between the cells. The wells were washed twice with PBS and then fulfilled with 500 µL of a fresh culture medium. Images of the wounded area containing migrated cells were taken at 0 and 24 h using an Olympus BX51 microscope, whereas measurements were performed using Image J.

Cell migration was quantified after determining the surface area of the defined wound area (total surface area) and migrated cells into the wounded area (migrated cell surface area): Percentclosure(%)=Migratedcellsurfacearea/Totalsurfacearea×100.

### Transwell invasion assay

The invasion of ESCC cells was evaluated with the 8 μm-pore Transwell (Corning, MA, USA). In brief, 1 × 10^5^ cells in 200 μL of the FBS-free medium were added to the matrigel-coated upper chamber, whereas 700 μL of the medium containing 10% FBS was added to the lower chambers. After 48 h incubation, non-invaded cells were removed by cotton swab, whereas invaded cells were fixed in 4% methanol for 30 min, dyed by 0.1% crystal violet (Beyotime) for 20 min, and counted under the Olympus BX51 microscope.

### Western blotting

The whole cell lysate was generated in a pre-cold radioimmunoprecipitation assay buffer. Protein concentration was measured using the BCA kit with the protocol provided by manufacturers. The protein extract was resolved on the 10–15% sodium dodecyl-sulfate polyacrylamide gel electrophoresis gel and transferred and electro-blotted onto a nitro-cellular membrane (Millipore). The membrane with transient wash was then blocked by incubation with 5% non-fat milk and incubated with primary antibodies at 4 °C for 16 h. Secondary antibodies with HRP conjugation were used for chemiluminescent signal determination. Immunoreactive signals were assessed using a kit of enhanced chemiluminescence chromogenic substrates (Bio-Rad, Hercules, CA, USA).

### Immunofluorescent staining

ESCC cells growing on glass slides were washed in PBS, fixed in immunofluorescent staining fixation (Beyotime) for 10 min, permeabilized with 0.5% Triton-X100 for 10 min, and blocked for 1 h at RT with 5% bovine serum albumin and incubated with primary antibodies at 4 °C overnight. Cells were rinsed three times in PBS and sequentially incubated with the secondary antibody for 1h at RT. Cells were finally washed in PBS to remove the antibody mixture. An anti-fade mounting medium was used with DAPI for samples. Fluorescence images were acquired using an Olympus BX51 microscope.

### Statistical analysis

Data are presented as means ± Standard Deviations (S.D.s). Statistical analysis was conducted with GraphPad Prism 8.4.2 software (San Diego, USA). A two-tailed unpaired Student's *t*-test was performed to compare the two groups. A one-way analysis of variance combined with Tukey's post-multiple tests was conducted to compare multiple groups.

## Results

### Elevated TMEM26 expression in ESCC

**Fig. 1:**
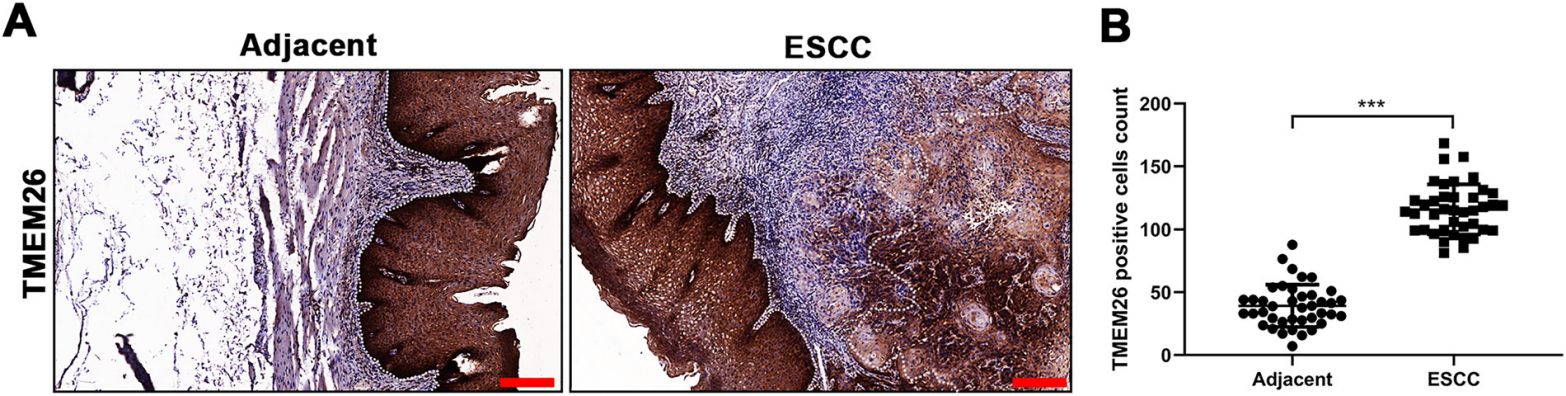
Elevated TMEM26 expression in ESCC. (A) Representative images of immunohistochemical staining for TMEM26 were conducted for slices from the ESCC tumor and control tissues from the same patient. (B) A higher number of TMEM26-positive cells were quantified in the ESCC tumor tissues compared with the control tissues from the same patient. Data are presented as mean ± S.D., *n* = 40, ***p < 0.001.

To determine the TMEM26 expression in human ESCC, immunohistochemical staining for TMEM26 was performed for slices from the tumor and adjacent normal tissues from patients with ESCC. Evidently, the tumor tissues exhibited higher staining (Fig. 1A) and contained a higher number of TMEM26-positive cells by quantification (Fig. 1B). This result was suggestive of a supportive role of TMEM26 in ESCC progression.

**Fig. 2:**
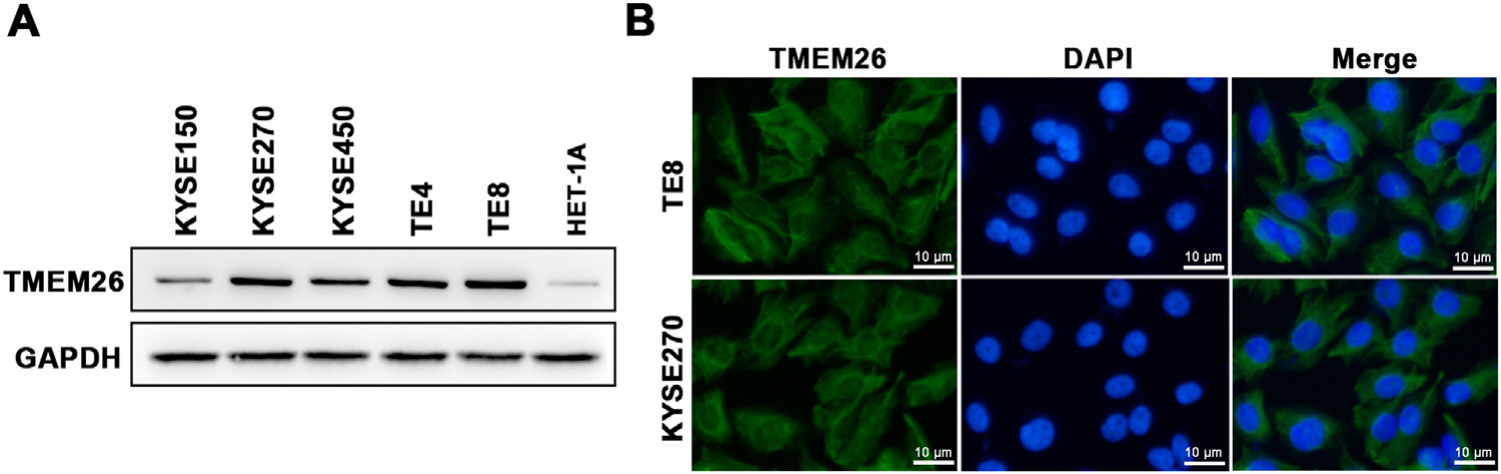
TMEM26 expression and cellular localization in various ESCC cell lines. (A) The abundance of TMEM26 in the normal esophageal epithelial cell line (HET-1A) and a collection of ESCC cell lines (KYSE150, KYSE270, KYSE450, TE4, and TE8) were examined by western blotting. (B) Immunofluorescent staining of TMEM26 with DAP in KYSE270 and TE8 cells verified the plasma membrane localization of TMEM26. Scale bar = 10 µm.

A collection of ESCC cell lines (KYSE150, KYSE270, KYSE450, TE4, and TE8) and a normal esophageal epithelial cell line (HET-1A) were examined with western blotting to assess the number of TMEM26. TMEM26 expression level was higher in all included ESCC cell lines than that in HET-1A cells, and a clear cell-to-cell variability was observed in TMEM26 expression (Fig. 2A). The KYSE270 and TE8 showed a higher number of TMEM26 and were selected to further examine TMEM26 depletion. Conversely, KYSE150, and KYSE450 showed a lower number of TMEM26 and were selected to further evaluate TMEM26 ectopic overexpression. An elevated TMEM26 expression in KYSE270 and TE8 cells was verified with immunofluorescent staining, where the cell membrane localization of TMEM26 was clearly observed (Fig. 2B).

### Silencing TMEM26 blocked the EMT-associated processes of ESCC

**Fig. 3:**
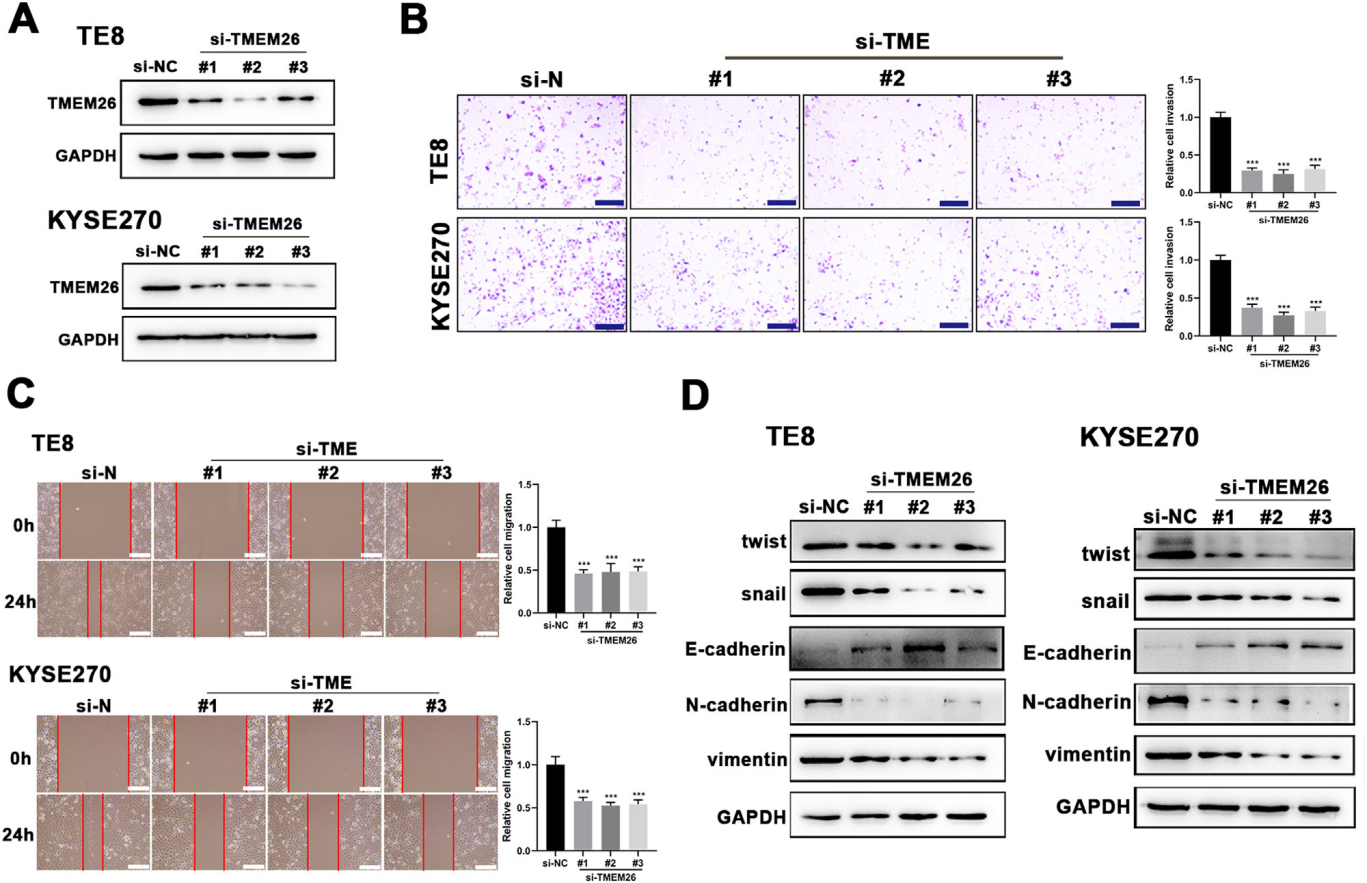
TMEM26 depletion in ESCC cells suppressed EMT-related alterations. (A) Transfection of siRNA targeting the TMEM26 gene (three independent targets) efficiently downregulated TMEM26 expression in TMEM26-high KYSE270 and TE8 cells, as detected in western blotting. (B) Transwell assay revealed a reduced number of invaded cells with TMEM26 RNAi, compared with control RNAi, in TMEM26-high KYSE270, and TE8 cells. Left: representative images. Right: quantification and statistical result. Scale bar = 100 µm. (C) Wound healing assay for TMEM26 RNAi KYSE270 and TE8 cells showed fewer cells migrating into the wound region, compared with control RNAi cells. Left: representative images. Right: quantification and statistical result. Scale bar = 100 µm. (D) Western blotting to detect mesenchymal and epithelial marker expressions showed downregulated twist, snail, N-cadherin, and vimentin but upregulated E-cadherin in TMEM26 RNAi KYSE270 and TE8 cells. Data are presented as means ± S.D., *n* = 3, ***p < 0.001.

Suspecting TMEM26 as a critical factor involved in the EMT of ESCC, the high TMEM26 expressions in ESCC cells, KYSE270, and TE8, were examined to assess the effects of TMEM26 depletion. Transfection of siRNA targeting TMEM26 gene (three independent targets) efficiently downregulated TMEM26 expression in these two cells, as detected in western blotting (Fig. 3A). In both KYSE270 and TE8, TMEM26 RNAi cells displayed altered invasion capability compared with control cells, i.e., lower number of invaded cells (Fig. 3B). Consistent with the Transwell assay, the wound healing assay for cells was performed to determine the capability of migration and invasion. In both KYSE270 and TE8, the migration of TMEM26 RNAi cells was slower than that of the control cells (Fig. 3C), which was supported by statistical analysis with repeated experiments (Fig. 3C). The changed morphology and migration and invasion ability could increase from reversed EMT; thus, marker proteins of EMT were examined using western blotting in TMEM26 RNAi or control KYSE270 and TE8 cells. Four mesenchymal markers (twist, snail, N-cadherin, and vimentin) were downregulated, whereas the epithelial marker (E-cadherin) was upregulated in TMEM26 RNAi KYSE270 and TE8 cells (Fig. 3D), indicating the reversed EMT process in TMEM26-depleting ESCC cells.

### TMEM26 overexpression aggravated EMT-associated processes of ESCC

**Fig. 4:**
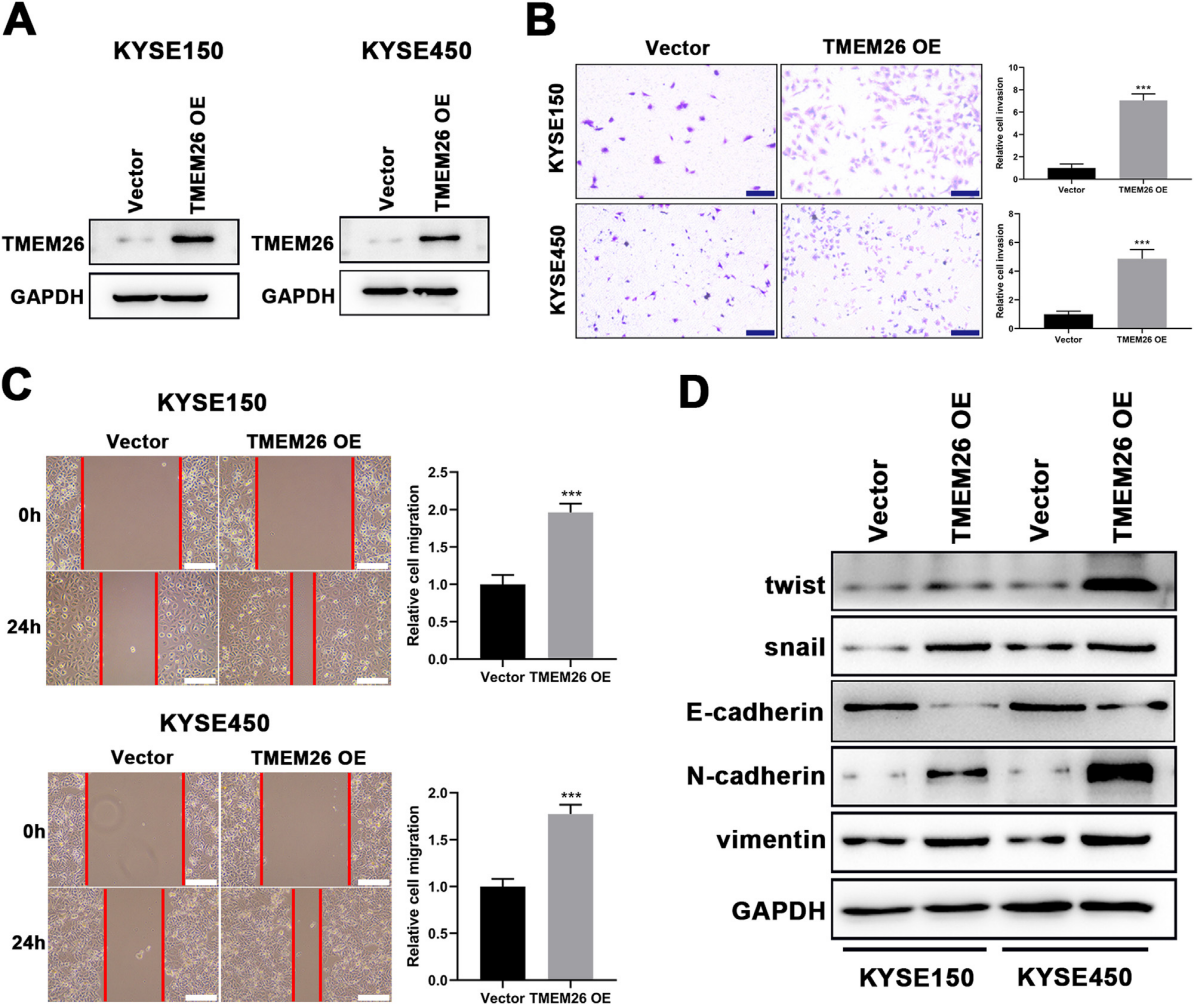
TMEM26 overexpression in ESCC cells promoted EMT-related alterations. (A) Transfection of TMEM26-expressing plasmid by lentiviral vectors efficiently over-regulated TMEM26 in TMEM26-low KYSE150 and KYSE450 cells, as shown by western blotting. (B) Transwell assay showed a higher number of invaded cells with TMEM26 overexpression than with control transfection in TMEM26-low KYSE150 and KYSE450 cells. Left: representative images. Right: quantification and statistical result. Scale bar = 100 µm. (C) The wound healing assay showed that the number of cells migrating into the wound region in TMEM26-overexpressing KYSE150 and KYSE450 cells was higher than that in control transfection cells. Left: representative images. Right: quantification and statistical result. (D) Western blotting detection of mesenchymal and epithelial marker expressions showed upregulated twist, snail, N-cadherin, and vimentin but downregulated E-cadherin in TMEM26-overexpressing KYSE150 and KYSE450 cells than that in control transfection cells. Data are presented as means ± S.D., n = 3, ***p < 0.001.

Consistent with findings from TMEM26 RNAi cells, the effects of TMEM26 overexpression in ESCC cells with high TMEM26 expression, KYSE150, and KYSE450, were also examined. Lentiviral transfection of the TMEM26 plasmid resulted in efficient TMEM26 overexpression in KYSE150 and KYSE450 cells, as shown by western blotting detection (Fig. 4A). In both KYSE150 and KYSE450, the invasive ability of TMEM26-overexpressing cells was stronger than that of control cells (Fig. 4B). Consistent with the observation of invasion, the wound healing assay for cells indicated that TMEM26-overexpressing cells in both KYSE150 and KYSE450 displayed accelerated migration compared with control cells (Fig. 4C), a finding supported by statistical analysis with repeated experiments (Fig. 4C). The EMT marker proteins were also examined by western blotting in TMEM26-overexpressing or control KYSE150 and KYSE450 cells. In the TMEM26-overexpressing cells, the expression of twist, snail, N-cadherin, and vimentin was significantly elevated, whereas that of E-cadherin was downregulated (Fig. 4D). Taken together, the number of TMEM26 determined the EMT process of ESCC cells, indicating that higher TMEM26 enhanced EMT.

**Fig. 5:**
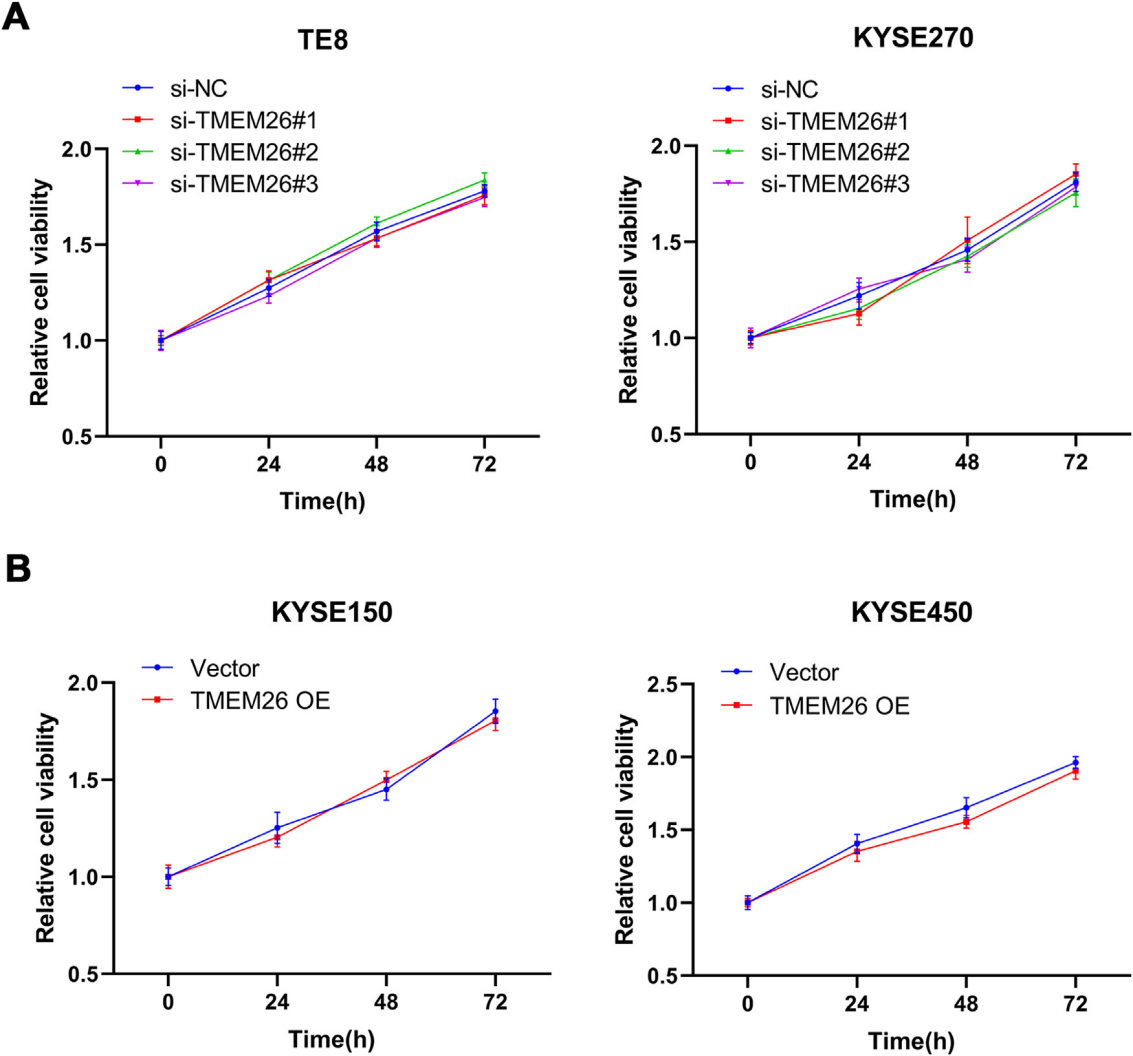
TMEM26 did not affect ESCC cell growth. (A) In TE8 (left) and KYSE270 (right) cells, siRNA knockdown of TMEM26 (three independent targets) did not show observable differences in the growth curve compared with siRNA control cells, as determined by MTT assay. (B) In KYSE150 (left) and KYSE450 (right) cells, TMEM26 overexpression due to lentiviral transfection did not show observable differences in the growth curve compared with control cells, as determined by MTT assay. Data are presented as means ± S.D., *n* = 6.

To understand whether TMEM26 is involved in ESCC cell proliferation that influences cell migration and invasion, an MTT assay was also carried out after TMEM26 RNAi or overexpression in ESCC cells. In KYSE270 and TE8, siRNA knockdown of TMEM26 (three independent targets) did not show an observable difference in the growth curve compared with siRNA control cells (Fig. 5A). Similarly, TMEM26 overexpression in KYSE150 and KYSE450 cells did not alter the growth rate of the cells (Fig. 5B). Therefore, TMEM26 did not contribute to the proliferation or death of ESCC cells, and its action in EMT should be considered differently.

### TMEM26 contributes to ESCC metastasis to the liver

**Fig. 6:**
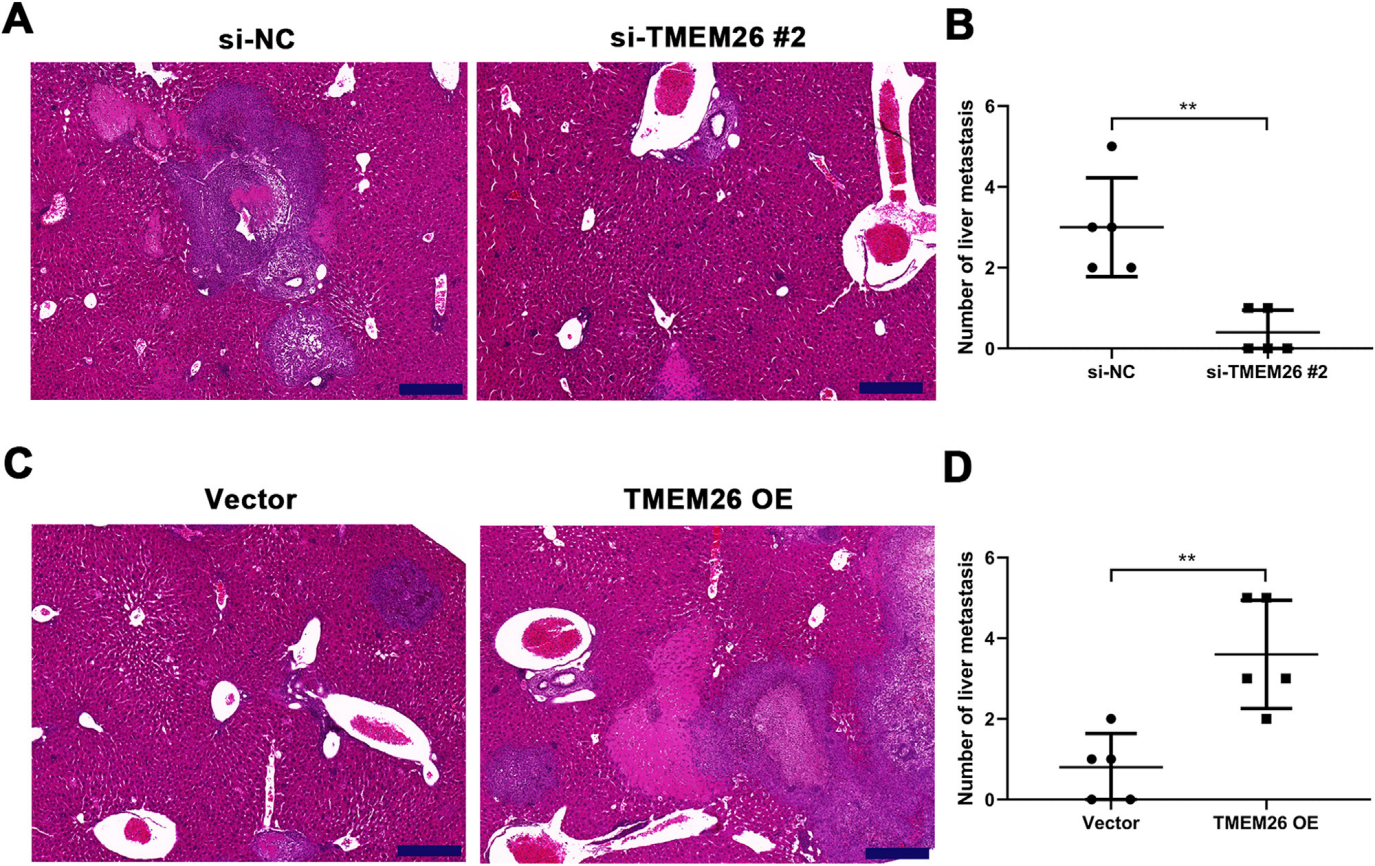
TMEM26 contributes to ESCC metastasis to the liver. (A) H&E staining showed a lower number of metastases and a reduced area of cancerous nests in the liver in the si-TMEM26#2 group. (B) H&E staining showed increased metastases and larger area of cancerous nests in the liver in the TMEM26 OE group. Data are presented as means ± S.D., *n* = 5, **p < 0.01.

Along with *in vitro* EMT-related assays, the role of TMEM26 in ESCC metastasis was monitored on the liver metastatic murine model. Consistent with the *in vitro* analysis, the aggressive ability of KYSE270 cells to the liver was weakened after TMEM26 RNAi, as indicated by the lower number of metastases and reduced area of cancerous nests in the liver compared with the control (Fig. 6A,B). Conversely, TMEM26-overexpressing KYSE150 cells exhibited a more aggressive ability to the liver, as indicated by a higher number of metastases and the larger area of cancerous nests in the liver compared with the control (Fig. 6C,D). These results further confirmed the essential role of TMEM26 in ESCC metastasis, which is mediated by EMT.

### TMEM26 promoted NF-κB signaling and is involved in the EMT of ESCC

**Fig. 7:**
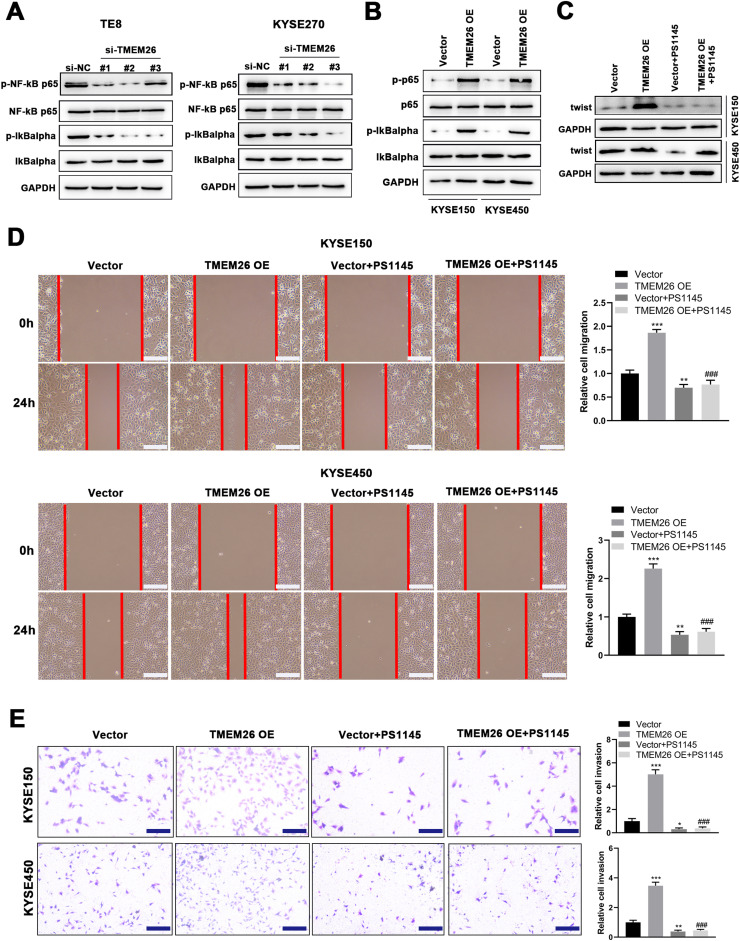
Detection for NF-κB signaling activation by TMEM26 and the determining effects of NF-κB signaling on EMT in ESCC cells. (A) In TMEM26-high TE8 (left) and KYSE270 (right) cells, siRNA knockdown of TMEM26 (three independent targets) did not show observable differences for the total number of p65 and IκBα; however, the p65 and IκBα phosphorylation was dramatically declined, as detected in western blotting. (B) TMEM26 overexpression in TMEM26-low KYSE150 and KYSE450 cells resulted in elevated p65 and IκBα phosphorylation, whereas the total amount of p65 and IκBα remained comparable, as detected in western blotting. (C) In KYSE150 and KYSE450 cells, TMEM26 overexpression enhanced the expression of twist; however, the treatment with PS1145, an NF-κB inhibitor, blocked this effect, as detected in western blotting. (D) Wound healing assay for TMEM26-overexpressing KYSE150 and KYSE450 cells showed a higher number of cells migrating into the wound region, which was blocked by PS1145. Left: representative images. Right: quantification and statistical result. Scale bar = 100 µm. (E) Transwell assay for KYSE150 and KYSE450 cells showed a higher number of invading cells after overexpressing TMEM26, which was blocked by PS1145. Right: quantification and statistical result. Scale bar = 100 µm. Data are presented as means ± S.D., *n* = 3, *p < 0.05, **p < 0.01, and ***p < 0.001 versus the vector group, ###*p* < 0.001 vs. the TMEM26 OE group.

Motivated by the report that NF-κB signaling was involved in the EMT of breast cancer cells.^20^, the present study examined the effects of TMEM26 on NF-κB signaling and whether this signaling mediates the action of TMEM26 in the EMT of ESCC cells. In TMEM26-overexpressing KYSE270 and TE8 cells, siRNA knockdown of TMEM26 (three independent targets) did not show an observable difference in the total number of p65 and IκBα, whereas the p65 and IκBα phosphorylation was dramatically declined, indicating an inhibited NF-κB signaling by TMEM26 knockdown (Fig. 7A). Consistent with this, TMEM26 overexpression in low TMEM26-expressing KYSE150 and KYSE450 cells resulted in elevated p65 and IκBα phosphorylation, whereas the total amount of p65 and IκBα remained comparable (Fig. 7B). PS1145 is a specific small-molecule IKK inhibitor used to inactivate NF-κB signaling in cancer cells.^21^ The treatment with PS1145 for KYSE150 and KYSE450 cells efficiently blocked the upregulation of twist expression through TMEM26 overexpression (Fig. 7C), demonstrating the indispensable role of NF-κB signaling in the EMT of ESCC cells. Corroborated with this, wound healing and Transwell assays showed that PS1145 intervention effectively blocked the TMEM26 overexpression-induced promotion in the migration and invasion of KYSE150 and KYSE450 cells (Fig. 7D and E).

### TMEM26 impaired TJ to support EMT of ESCC

**Fig. 8:**
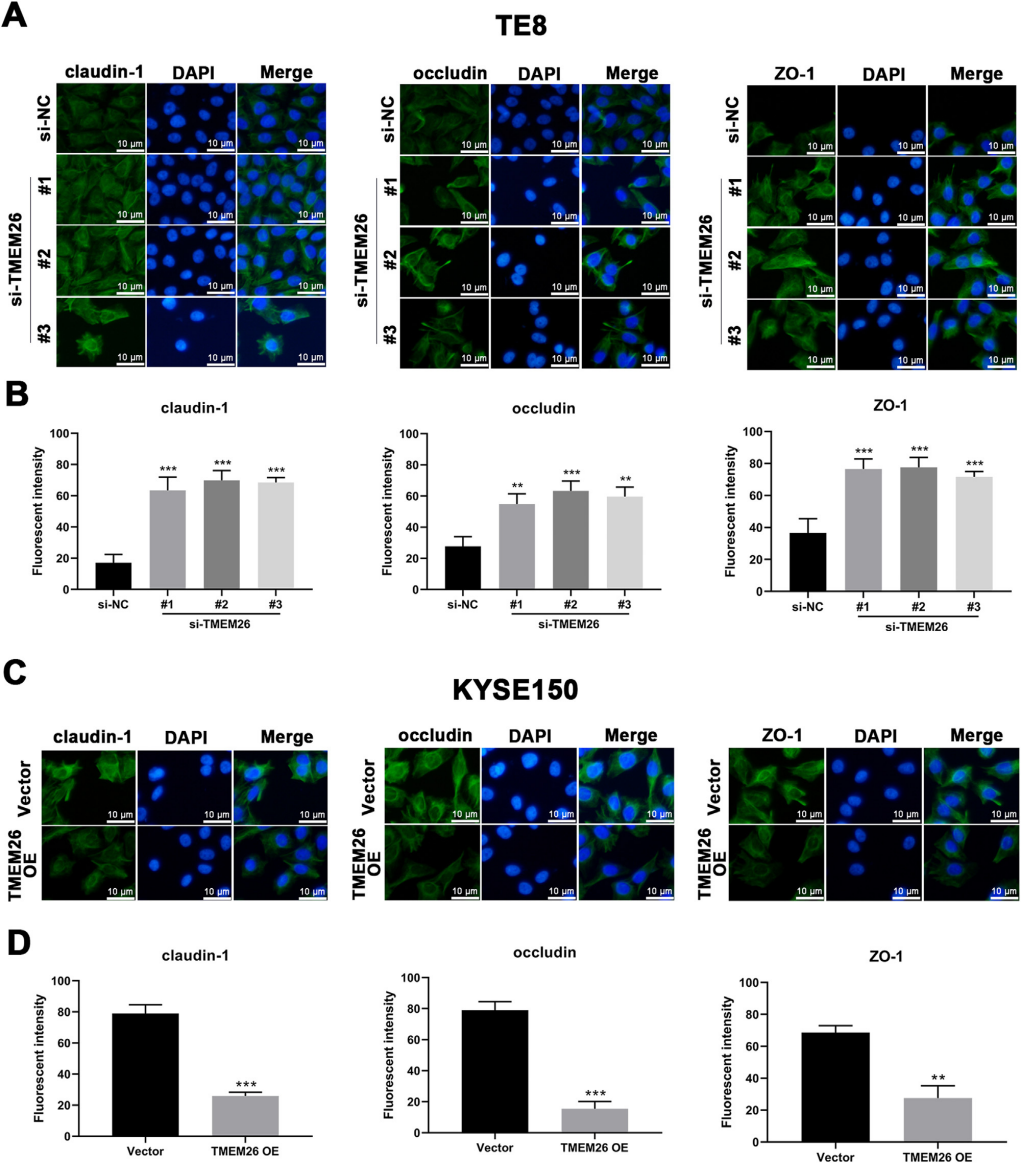
Regulation of plasma membrane distribution of TJ proteins by TMEM26 in TE8 and KYSE150 ESCC cells. (A and B) Immunofluorescent staining for claudin-1o Occludin, and ZO-1 with DAPI in TE8 cells showed their plasma membrane localization. Compared with the siRNA control, the TE8 cells with siRNA knockdown of TMEM26 (three independent targets) elevated the distribution of claudin-1, occludin, and ZO-1 at the plasma membrane. Quantification of plasma membrane signal intensity is shown in (B). (C and D) Immunofluorescent staining for claudin-1, occludin, and ZO-1 with DAPI in KYSE150 cells showed their plasma membrane localization. Compared with control transfection, KYSE150 cells with overexpressed TMEM26 showed a reduced distribution of claudin-1, occludin, and ZO-1 at the plasma membrane. Quantification of plasma membrane signal intensity is shown in (D). Scale bar = 10 µm. Data are presented as means ± S.D., *n* = 3, **p < 0.01, and ***p < 0.001.

In addition to the finding that TMEM26 activated the NF-κB signaling to promote EMT, exploring the direct role of TMEM26 in the TJ complex assembly at the plasma membrane would be beneficial, which would also affect the EMT process. Immunofluorescent staining for three TJ proteins, claudin-1, occludin, and ZO-1, with specific antibodies, confirmed their plasma membrane localization (Figs. 8 and S1). Compared with the siRNA control, the TE8 cells with siRNA knockdown of TMEM26 (three independent targets) demonstrated a significantly elevated intensity of claudin-1, occludin, and ZO-1 expression (Fig. 8A and B). In parallel, very similar, albeit weaker, effects of TMEM26 RNAi on the elevation of claudin-1, occludin, and ZO-1 signals were observed in KYSE270 cells (Fig. S1 A and B). Consistent with the findings from TE8 and KYSE270 cells, the TJ protein alteration was also observed in KYSE150 cells with TMEM26 overexpression. The intensity of claudin-1, occludin, and ZO-1 expressions was all significantly reduced (Fig. 8C and D). Consistently, TMEM26 overexpression in KYSE450 cells resulted in a lower intensity of claudin-1, occludin, and ZO-1 expressions (Fig. S1C and D). Taken together, TMEM26 directly interfered with the TJ complex assembly in ESCC cells, which could be an upstream factor of the EMT process.

## Discussion

As a devastating disease, esophageal cancer is the 6th most common cause of cancer deaths worldwide. China is experiencing an increasing health burden from EC, currently accounting for > 50% of the global cases.^22^ EC is classified into two subtypes with distinct histopathological features: ESCC and EAC. ESCC is more frequently diagnosed in East Asia, whereas EAC is more common in Western countries.^23^ Despite the rapid progress of surgery, chemotherapy, radiation therapy, molecular targeted therapy, and combinatorial therapies for ESCC, its prognosis remains poor, with an extremely low overall 5-year survival rate.^23^ The main limiting factor for efficient ESCC treatment is the high incidence rate of metastasis, including the lymph nodes, lungs, liver, bones, adrenal glands, and brain.^24^ Therefore, beneficial therapeutic outcomes can be achieved through understanding the molecular and cellular mechanisms involved in metastasis, particularly the EMT in ESCC.

To elucidate the EMT mechanisms in ESCC, this study focused on TMEM26, a novel gene encoding a putative plasma membrane protein with multiple transmembrane domains. By examining TMEM26 in the ESCC and para-tumor control tissues, TMEM26 expression was elevated in the ESCC tumor. In addition, a series of ESCC cell lines showed high TMEM26 expression, where its plasma membrane localization was confirmed. The critical role of TMEM26 in promoting EMT was indicated by the invasion in Transwell assay, migration in wound healing assay, and expression of mesenchymal marker genes in either TMEM26 silencing or overexpressing ESCC cells. Moreover, *in vivo* study was also conducted and verified the critical role of TMEM26 in ESCC metastasis. Interestingly, the cell growth did not exhibit a similar change with TMEM26 knockdown or overexpression, indicating distinct mechanisms for EMT and proliferation/cell death controls in ESCC cells.

A plethora of signaling pathways mediated the EMT of cancer cells, including the prototypical proinflammatory NF-κB signaling pathway. The nuclear translocation of NF-κB p65 can induce the transcription of several genes involved in EMT induction.^25^ The pterostilbene-isothiocyanate, a pterostilbene derivative, reverted the EMT in breast cancer metastatic cell line (MDA-MB-231) and *in vivo* 4T1-cell-induced metastatic mice model, by preventing the IKK complex, central to NF-κB activation.^26^ In normal breast epithelial and breast cancer cells, both IKK-β and NF-κB p65 were required for TNF-α-induced Twist1 expression, suggesting the involvement of canonical NF-κB signaling in EMT in these cells.^20^ Isoimperatorin, the *Angelica dahurica* derivative, inhibited cell migration, invasion, and proliferation by inactivating NF-κB signaling in colorectal and hepatocellular carcinoma cells.^27^ Cytidine monophospho-N-acetylneuraminic acid hydroxylase pseudogene, overexpressed in gastric cancer and associated with poor prognosis, enhanced the p65 expression to activate the NF-κB pathway, which then increased the amount of snail, the key transcription factor for EMT.^28^ Moreover, homeobox D11 has been reported to activate the NF-κB signaling pathway, thereby promoting the malignant characteristics of ESCC.^29^ Based on these previous findings, whether TMEM26 regulates the EMT in ESCC cells through the NF-κB pathway was determined. Detection of altered p65 and IκBα phosphorylation in ESCC cells with either TMEM26 knockdown or overexpression demonstrated the activating role of TMEM26 in the NF-κB pathway in ESCC cells. Notably, the treatment with PS1145, a specific small-molecule IKK inhibitor, efficiently blocked the promotion of EMT transcription factor expression, cell migration, and invasion induced by TMEM26, demonstrating the indispensable role of NF-κB signaling in the EMT of ESCC cells.

TJs and signaling pathways initiated by TJs are attractive targets for cancer treatments. Claudin 1 TJ protein has been recently reported to mediate RNA-binding protein LIN28B to promote invasion and liver metastasis of colorectal cancer.^30^ Therefore, TJ protein inhibitors and monoclonal antibodies may be employed to suppress EMT and metastasis.^13^ By observing the molecular architecture, most TJ complexes were found to contain membrane-attached cytoplasmic plaques that regulate junction assembly and were composed of multivalent scaffold proteins.^31^ Recently, TJ assembly at the plasma membrane was reportedly governed by phase separation, a process by which a well-mixed solution of macromolecules (e.g., TJ proteins) spontaneously separates into a dense and a dilute phase.^32^^,^^33^ Therefore, it should be determined whether TMEM26 exerts direct effects on TJ assembly at the plasma membrane. By examining multiple ESCC cells, with either TMEM26 knockdown or overexpression, TMEM26 effectively suppressed the plasma membrane presentation and TJ protein assembly.

Collectively, the present study determined the TMEM26 elevation in ESCC tumors and various ESCC cell lines, where the plasma membrane localization of TMEM26 was also confirmed. The RNAi depletion of TMEM26 in ESCC cells suppressed EMT-related alterations, including invasion, migration, and marker gene expression. Conversely, TMEM26 overexpression in ESCC cells promoted these EMT-related alterations. Mechanistically, TMEM26 promoted NF-κB signaling to accelerate EMT in ESCC cells. The plasma membrane presentation and TJ protein assembly were impaired by TMEM26.

In summary, the present study identified the important role of TMEM26, a lesser-known plasma membrane protein, in the EMT of ESCC cells for the first time. Although TMEM26 was elevated in ESCC, no genetic evidence supporting the importance of TMEM26 in ESCC has been published. Future studies will be required to identify the mutation, copy number variation alteration, and other types of genetic defects of TMEM26 in ESCC. Besides, the specific mechanism of how NF-κB signaling is regulated by TMEM26 has not been elucidated in this study. In the future, identifying the molecule-specific regulation between NF-κB signaling and TMEM26 is another important direction of the present study. Regarding the TJ assembly, the direct interaction between TMEM26 and TJ protein was not determined although the plasma membrane presentation and TJ protein assembly were impaired by TMEM26. Thus, future studies should evaluate the detailed mechanism of how TMEM26 impairs TJ assembly using biophysical methods (particularly phase separation assay in the cells and with purified proteins).

## Conclusion

In conclusion, the present study, for the first time, examined TMEM26 expression between tumorous and adjacent tissues from patients with ESC and demonstrated that TMEM26 expression was high in ESCC samples and cell lines. The present findings identified the promotion of TMEM26 on the EMT processes of ESCC cells *in vitro* and *in vivo*, which might rely on its role in activating NF-κB signaling and disrupting TJ formation. TMEM26 might be a potential therapeutic target for metastatic ESCC.

## Funding

This study was financially supported by the Science and Technology Plan Project of Suzhou (SZM2022009) and the Horizontal Research Foundation of Soochow University (P142900221).

## CRediT authorship contribution statement

**Guohu Han:** Conceptualization, Writing – original draft. **Shuangshuang Zhou:** Data curation. **Junjun Shen:** Data curation. **Yuanyuan Yang:** Data curation. **Xuyu Bian:** Formal analysis. **Yahu Li:** Formal analysis. **Rui Ling:** Formal analysis. **Rongrui Liang:** Conceptualization, Writing – review & editing. **Min Tao:** Conceptualization, Writing – review & editing.

## Declaration of Competing Interest

The authors declare no conflicts of interest.

## Appendix Supplementary materials

Image, application 1

Image, application 2

Image, image 3

Image, application 4
