# Supplementary material for: The role of TMEM26 in disrupting tight junctions and activating NF-κB signaling to promote epithelial-mesenchymal transition in esophageal squamous cell carcinoma
Source: Clinics (Sao Paulo). 2023 Aug 21;78:100276. doi: 10.1016/j.clinsp.2023.100276 (PMC10466919; doi:10.1016/j.clinsp.2023.100276)
Supplement: Supplementary file 1 [file mmc1.docx]

**Table S1. The information of antibodies**

| Antibody | Manufacturers | Cat.no | molecular weight | concentration |
| --- | --- | --- | --- | --- |
| TMEM26 | Thermo Fisher Scientific | PA5-23477 | 50 kDa | 1:1000 |
| twist | Beyotime | AF8274 | 20 kDa | 1:1500 |
| snail | Beyotime | AF8013 | 29 kDa | 1:1200 |
| N-cadherin | Beyotime | AF0243 | 140 kDa | 1:800 |
| vimentin | Beyotime | AF1975 | 54 kDa | 1:2000 |
| p-NF-kB p65 | Abcam | ab183559 | 80 kDa | 1:1000 |
| NF-kB p65 | Abcam | ab32536 | 65 kDa | 1:1500 |
| p-IkBalpha | Abcam | ab133462 | 40 kDa | 1:10000 |
| IkBalpha | Abcam | ab32518 | 35 kDa | 1:2000 |
| GAPDH | Beyotime | AF1186 | 36 kDa | 1:2500 |
| ZO-1 | Beyotime | AF8394 | 250 kDa | 1:100 |
| claudin-1 | Beyotime | AF6504 | 20 kDa | 1:80 |
| occludin | Beyotime | AF7644 | 59 kDa | 1:100 |
