# Supplementary material for: The role of TMEM26 in disrupting tight junctions and activating NF-κB signaling to promote epithelial-mesenchymal transition in esophageal squamous cell carcinoma
Source: Clinics (Sao Paulo). 2023 Aug 21;78:100276. doi: 10.1016/j.clinsp.2023.100276 (PMC10466919; doi:10.1016/j.clinsp.2023.100276)
Supplement: Supplementary file 2 [file mmc2.docx]

**Table S2. The information of sequences**

|  | Sequences | |
| --- | --- | --- |
| TMEM26-sh-1 | 5’-3’ | ACCTCGGACATTGGGACTCCATCAGATCAAGAGTCTGATGGAGTCCCAATGTCC |
| TMEM26-sh-2 | 5’-3’ | ACCTCGCTGACATACTGGAATTCACATCAAGAGTGTGAATTCCAGTATGTCAGC |
| TMEM26-sh-3 | 5’-3’ | ACCTCGTCTATGCCATCCTTGTTATATCAAGAGTATAACAAGGATGGCATAGAC |
| sh-NC | 5’-3’ | GAATGTCGGCAGGCCTAATCA |
