# Supplementary figures and images for: The role of TMEM26 in disrupting tight junctions and activating NF-κB signaling to promote epithelial-mesenchymal transition in esophageal squamous cell carcinoma
Source: Clinics (Sao Paulo). 2023 Aug 21;78:100276. doi: 10.1016/j.clinsp.2023.100276 (PMC10466919; doi:10.1016/j.clinsp.2023.100276)

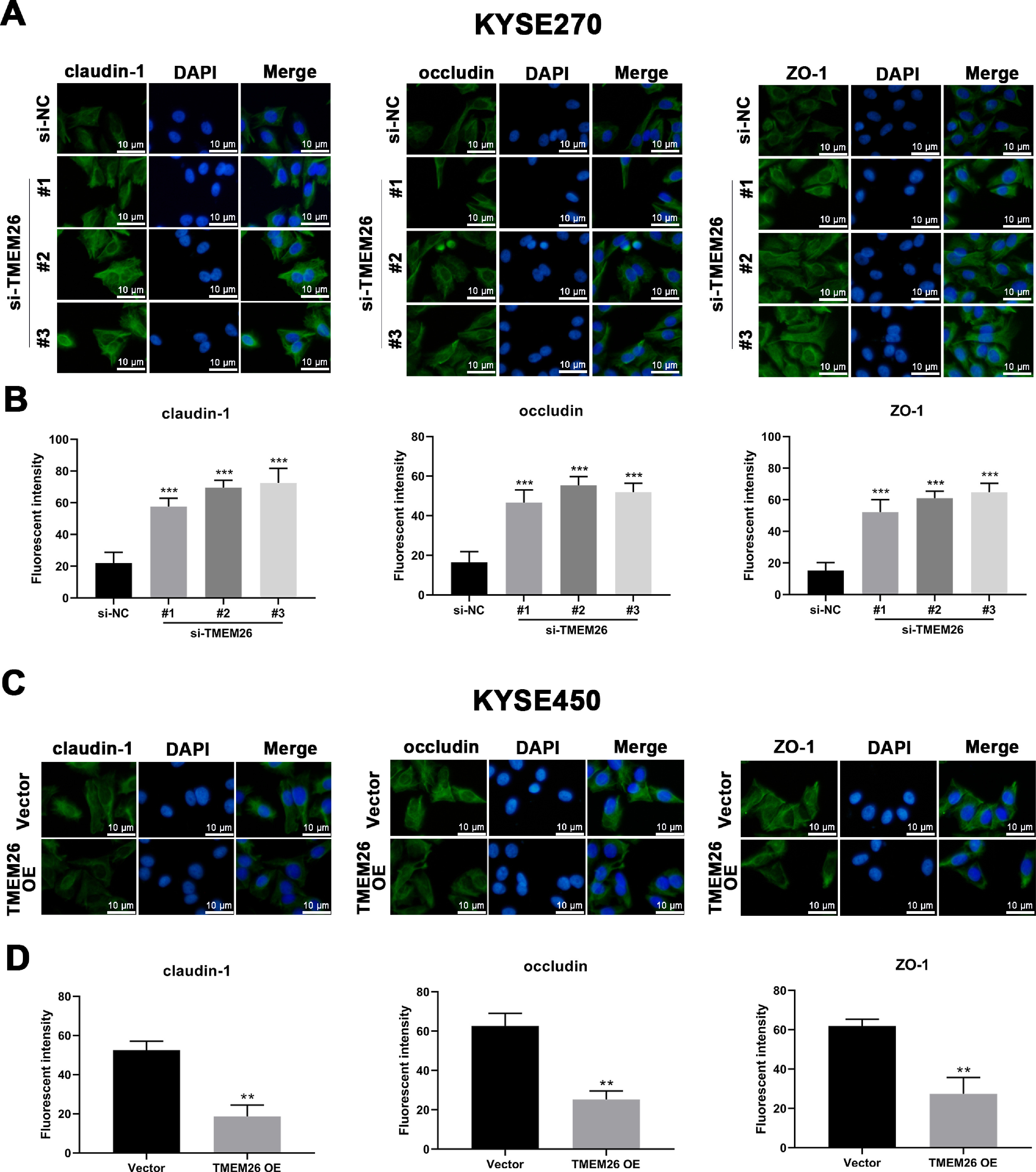

Supplement: Supplementary file 3 [file mmc3.jpg]
