# Supplementary material for: The role of TMEM26 in disrupting tight junctions and activating NF-κB signaling to promote epithelial-mesenchymal transition in esophageal squamous cell carcinoma
Source: Clinics (Sao Paulo). 2023 Aug 21;78:100276. doi: 10.1016/j.clinsp.2023.100276 (PMC10466919; doi:10.1016/j.clinsp.2023.100276)
Supplement: Supplementary file 4 [file mmc4.pdf]

enago
Author First, Quality First
www.enago.cn

CERTIFICATE OF EDITING

This is to certify that the paper titled The role of TMEM26 in disrupting tight junctions and activating NF-κB signaling to promote epithelial-mesenchymal transition in esophageal squamous cell carcinoma commissioned to us by Guohu Han, Shuangshuang Zhou, Junjun Shen, Yuanyuan Yang, Xuyu Bian, Yahu Li, Rui Ling, Rongrui Liang Min Tao has been edited for English language, grammar, punctuation, and spelling by Enago, the editing brand of Crimson Interactive Consulting Co., Ltd.

ISO 17100:2015 Translation Service Providers

ISO 27001:2013 Information Security Management System

ISO 9001:2015 Quality Management System

CRIMSON INTERACTIVE CONSULTING CO.,LTD 克里门森互动(北京)顾问有限公司 1101030093948

Issued by:

Enago, Crimson Interactive (Beijing) Consulting Co., Ltd.Room 3217,
Cyber Tower A,
No. 2, Zhongguancun South Street,Haidian
District, Beijing

Disclaimer: The intent of the author's message has been preserved during the editing process. The author is free to accept or reject our changes in the document after reviewing our editing. This certificate has been awarded at the time of sharing the final edited version (full file or sections of the file) with the author. Enago does not bear any responsibility for any alterations done by the author to the edited document post **18th July 2023**
